# Elevated Functional Connectivity in a Striatal-Amygdala Circuit in Pathological Gamblers

**DOI:** 10.1371/journal.pone.0074353

**Published:** 2013-09-02

**Authors:** Jan Peters, Stephan Franz Miedl, Christian Büchel

**Affiliations:** 1 Department of Systems Neuroscience, University Medical Center Hamburg-Eppendorf, Hamburg, Germany; 2 Helen Wills Neuroscience Institute, University of California, Berkeley, California, United States of America; 3 Department of Psychology, University of Salzburg, Salzburg, Austria; University College London, United Kingdom

## Abstract

Both substance-based addiction and behavioural impulse control disorders (ICDs) have been associated with dysfunctions of the ventral striatum. Recent studies using functional connectivity techniques have revealed increased coupling of the ventral striatum with other limbic regions such as amygdala and orbitofrontal cortex in patients with substance abuse disorders and attention-deficit hyperactivity disorder. In the present study, we re-analyzed previously published functional magnetic resonance imaging data acquired in pathological gamblers and controls during value-based decision-making to investigate whether PG is associated with similar functional connectivity effects. In line with previous studies in other ICDs, we observed reliable increases in functional coupling between striatum and bilateral amygdala in gamblers vs. controls. Implications of these findings for neural models of self-control and addiction are discussed.

## Introduction

Impulse control disorders (ICDs) including substance abuse are associated with impairments in value-based decision-making [1], [2], and non-substance based addictions such as pathological gambling (PG) share many features with substance-based addictions [3]. One of the most extensively-studied behavioural-economic correlates of ICDs is increased temporal discounting [2], [4], [5], i.e. an increased propensity to prefer smaller-but-sooner (SS) over larger-but-later (LL) rewards [6]. Pathological gamblers show increased temporal reward discounting compared to control participants [7]–[11] and also have the tendency for increased risk-taking [11]–[13].

The neural mechanisms underlying these problematic alterations in decision-making in gamblers are debated, but one candidate mechanism is striatal dysfunction [14], which is also implicated in substance abuse [1]. A number of functional magnetic resonance imaging studies in pathological and problem gamblers have revealed modulations in striatal and medial prefrontal responses during the processing of reward information [11], [15]–[21]. Taken together, however, these findings are inconsistent with a simple model in which PG is associated with generally elevated or diminished reward-related responses. Rather, factors such as the analyzed task phase (e.g. reward anticipation vs. outcome), analysis procedure (e.g. model-based vs. categorical analysis), gambling-relatedness of task and stimuli and reward type (e.g. primary vs. secondary reinforcers) jointly appear to influence whether PGs show elevated or attenuated striatal signals [11], [22]–[25].

In addition to examining such task-related neural activations [15]–[17], or parametric neural responses [11], [26], a complementary approach is to examine functional connectivity (i.e. time series correlations) between regions in relation to ICDs. Functional connectivity between the striatum, amygdala and orbitofrontal cortex is increased in drug addiction and attention-deficit hyperactivity disorder [27]–[29]. Ventral striatum and medial orbiotofrontal cortex/ventromedial prefrontal cortex (mOFC/vmPFC) are part of the limbic loop of the thalamo-cortico-basal-ganglia circuit [30], a loop that also has dense interconnections with hippocampus and amygdala [31]. But whether PG is associated with similar functional connectivity effects is unclear.

Interactions between striatum and amygdala are of particular interest in the context of reward processing. Animal work has consistently pointed towards an important role of functional interactions between striatum and amygdala in regulating reward-guided behaviour [32]–[34]. In the light of previous findings in other ICDs [27]–[29] it is therefore important to assess increased functional connectivity in this circuit in PGs, in order to determine whether this may constitute a mechanism underlying ICDs in general.

One previous study on functional connectivity in PGs focussed on response inhibition tasks rather than reward-based decision-making [35]. To address the role of functional connectivity in pathological gamblers during decision-making, we re-analyzed previously published data in controls and pathological gamblers performing two different value-based decision-making tasks during fMRI [11]: delay discounting (i.e. choosing between smaller-sooner and larger-later rewards) and probability discounting (i.e. choosing between smaller-certain and larger-riskier rewards). To directly assess the limbic basal-ganglia-thalamocortical circuit [14], [30], we focussed on group differences in functional connectivity with the left and right ventral striatal seed voxels.

## Methods

### General Overview

This study constitutes a re-analysis of a previously published functional magnetic resonance imaging (fMRI) data set [11] using functional connectivity analyses. All participants provided informed written consent, and the study procedure was approved by the local institutional review board (Ethics Committee of the Hamburg Board of Physicians). For a detailed description of the sample characteristics, task procedures, and fMRI data acquisition parameters please refer to Miedl et al. (2012). In short, participants (n = 16 pathological gamblers and n = 16 age- and education-matched control subjects) performed a value-based decision-making task during fMRI. Two randomly intermixed conditions were included: delay discounting and probability discounting [26]. In delay discounting trials, subjects made choices between 20’ available now and larger but delayed rewards. In probability discounting trials, subjects made repeated choices between 20’ with 100% probability and larger amounts with lower probabilities. The immediate/certain option of 20’ was not displayed. Trial timing was as follows: 1) 500 m fixation, 2) 2500 ms presentation of the delayed/risky option, 3) 3000–7000 ms jitter (drawn from uniform distribution), 4) max. 2500 ms choice screen (accept/reject delayed/risky option), 5) 3000–7000 ms jitter (drawn from uniform distribution).

We fit hyperbolic models of delay and probability discounting [6], [26] to each participant’s choice data using maximum-likelihood techniques [11]. Based on these single-subject model fits, we then calculated estimates of the subjective value of the larger-later (delay discounting) or the larger-riskier (probability discounting) reward on each trial. These estimates were then included as parametric regressors in a model-based fMRI analysis [36].

Trials for the fMRI task were generated in a subject-specific manner based on a behavioural pre-test. As PGs are characterized by excessively steep reward discounting, larger amounts of LL rewards are required for PGs compared to controls to obtain similar subjective reward values. The procedure of computing subject-specific offers ensured that subjective reward values were similar in the PG and control groups.

### FMRI Preprocessing

Preprocessing was carried out using SPM08 (Wellcome Department of Cognitive Neurology, University College London). Scans were slice-time corrected to the onset of the middle slice, and realigned to the mean functional scan using a 6-parameter affine transformation. Functional images were then co-registered with the high-resolution anatomical T1-weighted image. The anatomical scan was then segmented into grey matter, white matter, and CSF. Functional images were normalized to MNI space using the normalization parameters obtained from the segmentation procedure, and finally smoothed using an 8 mm full-width-half-maximum Gaussian kernel.

### Functional Connectivity Analyses

We used psycho-physiological interaction analyses (PPI) [37] as implemented in SPM08 to examine group differences in functional connectivity. For the connectivity analysis, delay and probability trials were modelled as 8s mini-blocks. From this first level model, seed time courses were extracted using the Volume of Interest (VOI) function of SPM08. Seeds were placed in the left and right ventral striatum at peaks from the main effect of subjective value (SV) across conditions and groups (i.e. striatal peaks showing a positive correlation with SV[larger later] and SV[risky option] across controls and gamblers).

For each seed region, a first level PPI model was set up including the following user-specified regressors: 1) the time course of the seed region, 2) a regressor coding for the experimental condition (1: delay discounting, −1: probability discounting), 3) the interaction term, i.e. the multiplication of regressors 1 and 2, and 4) the six parameters from the realignment procedure modelling movement-related effects. Single-subject contrast images for each of these three regressors were created. Because we were primarily interested in overall group differences in striatal connectivity, rather than group x condition interaction effects in connectivity, we took the contrast image for the striatal timecourse regressor to a second-level random-effects analysis (two sample t-test model). In keeping with our previous analysis [11] we included gambling severity [38], smoking severity [39] and depression scores [40] as group-specific covariates in this second-level model. Separate analyses were carried out for the seed in the left striatum, and for the seed in the right striatum.

In an additional control analysis, we extracted the seed time course in a condition-specific manner, i.e. separately for delay and probability trials. This allowed us to assess potential group x task interaction and conjunction effects. A first level PPI model was set up for each subject for this analysis using the following regressors: 1) the time course of the seed region during delay discounting trials, 2) a regressor coding for delay discounting trials (1 for delay discounting trials, 0 otherwise), 3) the time course of the seed region during probability discounting trials, 4) a regressor coding for probability discounting trials (1 for probability trials, 0 otherwise), and 5) the six parameters from the realignment procedure modelling movement-related effects. The contrast images for each striatal time course correlation (one contrast image for the seed timecourse during delay discounting, probability discounting) were taken to a second-level random-effects analysis in a 2 (controls/gamblers) x 2(delay/probability) full factorial model. We tested striato-amygdala coupling additionally in a conservative conjunction analysis [41], i.e. coupling gamblers>controls in delay discounting AND coupling gamblers>controls in probability discounting.

### Correction for Multiple Comparisons

Correction for multiple comparisons was performed using small-volume-correction (p_FWE_<0.05). For the ventral striatum we used 8 mm spheres centered at 14±10 −10 [42]. For the amygdala we used unilateral anatomical masks from the FSL software package (50% probability threshold).

## Results

### Main Effect of Subjective Value Across Groups and Conditions

**Figure 1:**
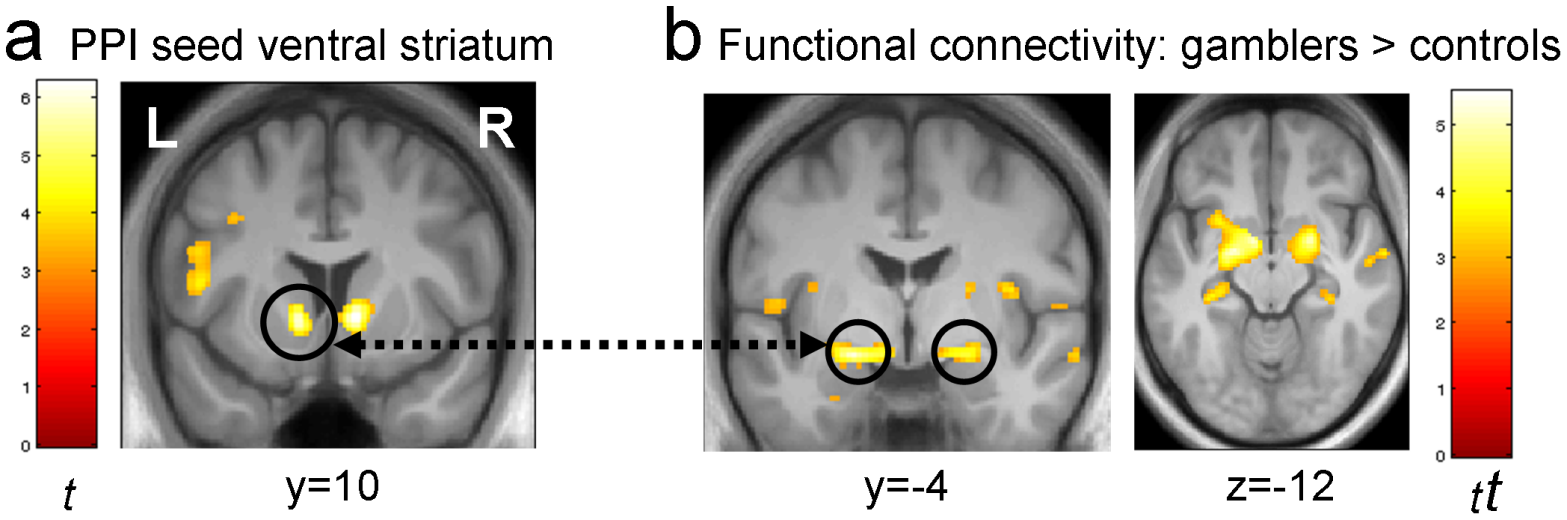
Results from the functional connectivity analysis in controls and pathological gamblers. a) A re-analysis of previously published fMRI data of decision-making in controls and gamblers revealed a main effect of subjective value across groups (controls and pathological gamblers) and conditions (delay and probability discounting). b) Functional connectivity of the left ventral striatum with bilateral amygdala was enhanced in the gamblers compared to the controls (left amygdala: p_svc_ = .001, right amygdala: p_svc_ = .005). Similar effects were seen when the seed was placed in the right ventral striatum (see Table 2). Left panels: display threshold p<0.001 uncorrected, 10 voxels (a) and p<0.005 uncorrected, 10 voxels (b).

In order to identify an unbiased ventral striatal activation peak as a seed region for the functional connectivity analyses, we first carried out a parametric analysis of subjective value of the delayed or risky option. We set up a second-level factorial model with the factors group (gamblers/controls) and task (delay discounting/probability discounting). The model included gambling severity [38], smoking behaviour [39] and depression [40] as group-specific covariates, as in our previous report [11]. Within this model, we now searched for regions showing a positive correlation with the value of the delayed/risky option across both groups and tasks (i.e. an overall main effect of subjective value; contrast vector [1 1 1 1]). The strongest activation in this contrast was localized in bilateral ventral striatum (Figure 1a, right: x,y,z coordinates 10, 10, −2, z-value = 5.41, p_svc_<.001, left: −10, 9, −9, z-value = 5.17, p_svc_<.001). These two striatal peaks were subsequently used as seed regions for the functional connectivity analysis.

### Group Differences in Striatal Functional Connectivity

**Figure 2:**
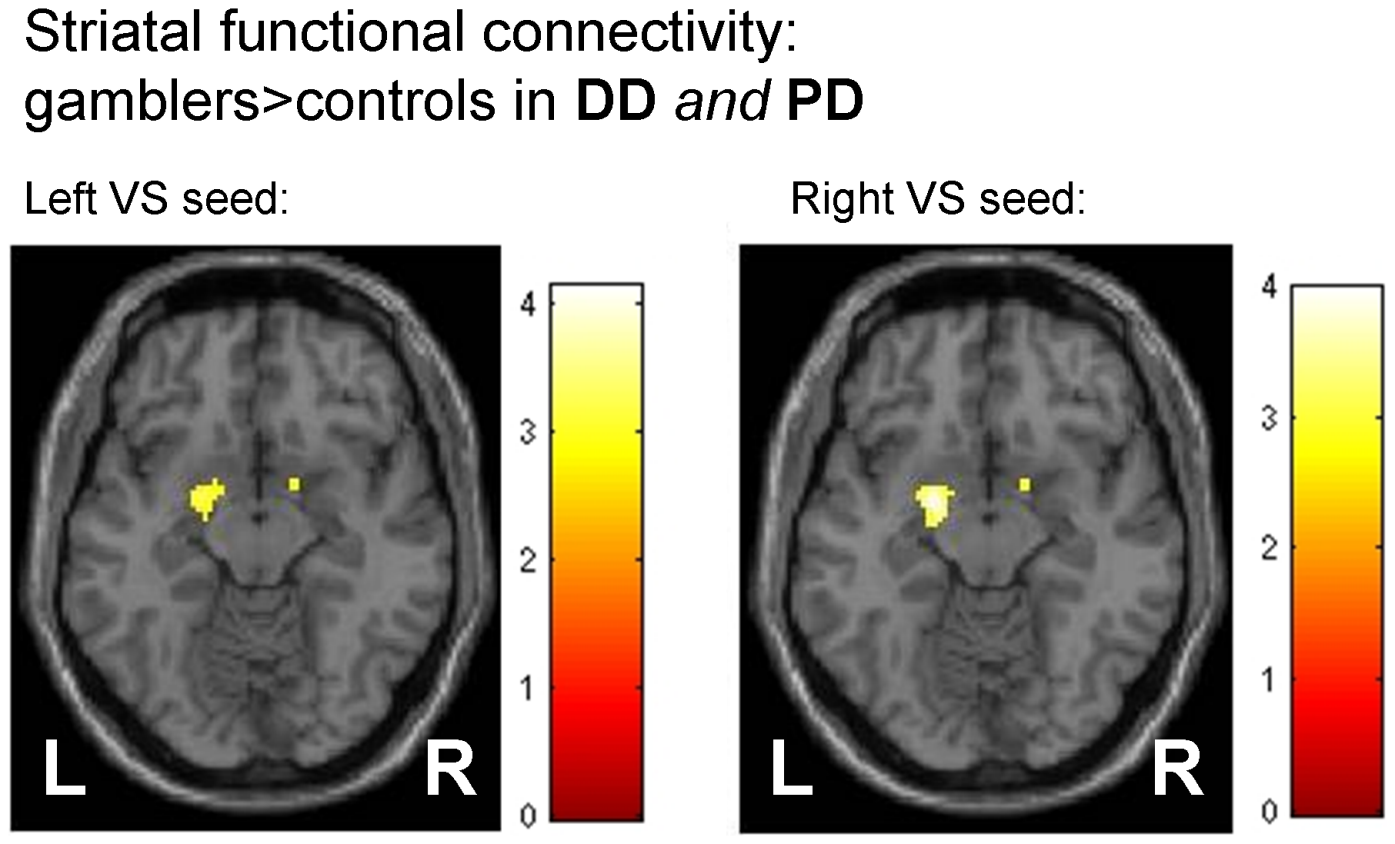
Regions showing greater striatal functional connectivity in gamblers vs. controls, conjunction analysis across delay and probability discounting trials (display threshold p<0.005 uncorrected for each contrast, p_svc_<.05 for left amygdala). Color bars denote t-values.

We carried out two analyses. First, we analyzed striatal connectivity across the entire session. Regions showing greater functional connectivity with left striatum in gamblers vs. controls are listed in Table 1. Regions from the same analysis for right ventral striatum are listed in Table 2. For the left striatal analysis, regions including bilateral amygdala showed strong increases in striatal coupling for gamblers vs. controls (Figure 2b, left amygdala: −20, −4, −12, z-value = 4.17, p_svc_ = .001, right amygdala: 22, −2, −12, z-value = 3.79, p_svc_ = .005). Similar increases were seen in vmPFC (−12, 40, 12, z-value = 3.62). As can be seen from Table 2, these same regions showed increased coupling with the right ventral striatum in gamblers vs. controls. No regions showed greater functional connectivity with left or right striatum in controls vs. gamblers at p<0.001 uncorrected. We did not observe associations between the degree of striatal-amygdala connectivity and inter-individual differences in delay/probability discounting or gambling severity within the gamblers group.

**Table 1:** Anatomical Site, Cluster Size, maximum Z value, and MNI coordinates of the local maxima for the connectivity analysis (seed in left ventral striatum [MNI coordinates −10 9 −9], connectivity gamblers>controls).

Region	cluster-size	Z	MNI-coordinates
R Superior/MiddleTemporal Gyrus	129	4.38	58 −24 −4
		3.86	64 −32 4
		3.26	52 −12 −10
L Amygdala/Lentiform nucleus	194	4.19	−8 0 −10
		4.17	−20 −4 −12
R Amygdala	73	4.06	22 0 −12
L Superior Temporal Gyrus	40	3.78	−40 −52 22
L Insula	46	3.78	−46 6 −2
L Anterior Cingulate/vmPFC	39	3.62	−12 40 12
L Precentral Gyrus	20	3.53	−52 −12 10
L Middle Temporal Gyrus	21	3.48	−64 −28 0
L Parahippocampal Gyrus	67	3.44	−16 −30 −6
		3.32	−24 −26 −8
		3.28	−30 −24 −16
R Precuneus	14	3.32	4 −54 32
L Inferior Frontal Gyrus	10	3.25	−28 10 −12
R Posterior Cingulate Gyrus	10	3.25	2 −42 30

Clusters were thresholded at p<0.001 uncorrected and a minimum size of 10 voxels.

**Table 2:** Anatomical Site, Cluster Size, maximum Z value, and MNI coordinates of the local maxima for the connectivity analysis (seed in right ventral striatum [MNI coordinates 10 10 −2], connectivity gamblers>controls).

Region	cluster-size	Z	MNI-coordinates
L Amygdala	261	4.64	−18 −4 −12
L Inferior Frontal Gyrus		3.73	−30 12 −12
R Parahippocampal Gyrus	62	4.43	28 −32 −12
R Amygdala	78	4.25	20 4 −12
L Parahippocampal Gyrus	196	4.12	−14 −40 −6
		4.05	−22 −26 −8
		3.29	−30 −34 −6
R Superior Temporal Gyrus	43	4.04	56 −26 −2
L Middle Temporal Gyrus	27	3.84	−64 −30 0
R Superior Temporal Gyrus	38	3.71	40 −48 20
R Posterior Cingulate Gyrus	17	3.54	4 −36 28
L Insula	33	3.5	−30 10 8
L Parahippocampal Gyrus	11	3.38	−24 −32 −20
L Cuneus	10	3.36	−28 −88 22

Clusters were thresholded at p<0.001 uncorrected and a minimum size of 10 voxels.

We next examined a model in which the seed time course was extracted in a condition-specific manner (see methods), which allowed us to examine group x task interaction effects. We obtained separate contrast images reflecting the degree of striatal functional connectivity for delay trials and probability trials, again separately for left and right striatal seeds. These images were taken to a second-level random effects analysis using a group (controls vs. gamblers) x task (delay vs. probability discounting) factorial model. Covariates were identical to the previous model. Tables 3 and 4 show results from the main effect of group (striatal connectivity gamblers>controls) again for left and right striatal seeds, respectively. As in the previous analysis, we observed an increase in striatal-amygdala functional connectivity in gamblers vs. controls (p_svc_<.05 for both left and right amygdala). This effect was also significant in the left amygdala (p_svc_<.05) when a conservative conjunction analysis [41] across both delay and probability trials was examined (Figure 2). No regions showed a significant group x task interaction at p<0.001 uncorrected.

**Table 3:** Anatomical Site, Cluster Size, maximum Z value, and MNI coordinates of the local maxima for the connectivity analysis (seed in right ventral striatum [MNI coordinates 10 10 −2], connectivity gamblers>controls, main effect across delay and probability discounting trials).

Region	cluster-size	Z	MNI coordinates
L Amygdala	85	4.03	−18 −4 −12
R Superior Temporal Gyrus	38	3.92	60 −24 −4
R Superior Temporal Gyrus	37	3.86	40 −48 20
R Posterior Cingulate	56	3.72	4 −38 26
R Ventral Striatum	46	3.64	16 12 −6
L Anterior Cingulate/vmPFC	38	3.63	−14 44 6
L Insula	59	3.62	−34 6 8
L Ventral Striatum	26	3.5	−18 12 −6
L Middle Temporal Gyrus	15	3.46	−68 −30 0

Clusters were thresholded at p<0.001 uncorrected and a minimum size of 10 voxels.

**Table 4:** Anatomical Site, Cluster Size, maximum Z value, and MNI coordinates of the local maxima for the connectivity analysis (seed in left ventral striatum [MNI coordinates −10 9 −9], connectivity gamblers>controls, main effect across delay and probability discounting trials).

Region	cluster-size	Z	MNI-coordinates
L Anterior Cingulate/vmPFC	122	4.17	−12 40 10
		3.29	−12 50 12
L Ventral Striatum	52	3.97	−14 10 −6
L Insula	176	3.92	−36 2 8
		3.72	−32 12 8
		3.34	−34 −12 14
R Inferior Parietal Lobule	24	3.58	44 −48 22
R Inferior Frontal Gyrus	41	3.53	48 34 −2
L Inferior Frontal Gyrus	66	3.52	−48 14 −6
		3.46	−48 6 −2
		3.22	−52 −2 4
R Superior Temporal Gyrus	42	3.49	58 −50 12
R Superior Temporal Gyrus	18	3.45	58 −24 −4
R Caudate	29	3.43	22 −20 24
L Superior Temporal Gyrus	31	3.4	−42 −54 22
		3.39	−50 −58 22
L Amygdala	23	3.4	−20 −2 −12
L Middle Temporal Gyrus	14	3.34	−68 −30 2
R Superior Temporal Gyrus	11	3.33	56 −36 4

Clusters were thresholded at p<0.001 uncorrected and a minimum size of 10 voxels.

Finally, we examined whether the observed effects in the gamblers were attributable to an increase in positive connectivity or a decrease in negative connectivity. We therefore carried out a triple conjunction analysis in the previous factorial model, searching for regions showing 1) a positive correlation with the striatum in the control group, 2) a positive correlation with the striatum in gamblers and 3) a greater effect gamblers than controls. This analysis yielded again activity in the amygdala (−18, −2, −12, z-value = 3.97 [right striatal seed], −20, −2, −12, z-value = 3.40 [left striatal seed]), showing that positive striatal-amygdala connectivity in the gamblers was elevated.

## Discussion

Addiction has been consistently linked to ventral striatal dysfunction [1]. Pathological gambling shares many features with substance-based addiction, and reward-related responses in striatal and frontal regions consistently show modulations in gamblers compared to control subjects [11], [14]–[21]. One approach that may provide additional insight in the neurobiological mechanisms underlying such impulse control disorders (ICDs) are analyses of functional connectivity [43]. In resting state functional connectivity studies, correlations between the time series of different brain regions are examined in the absence of a cognitive task. Striatal resting state connectivity has previously been associated with ICDs, e.g. in children with ADHD [27], heroin addicts [28], [29] and smokers [44].

Alternatively, as in the present study, group differences in functional connectivity can also be examined performance of a cognitive task [35]. This previous study examined differences in functional connectivity between controls and problem gamblers during a response inhibition paradigm [35]. In contrast, we examined differences in striatal functional connectivity during performance of two difference value-based decision-making tasks (delay and probability discounting), in a re-analysis of previously published data [11]. One advantage of analyzing task-related vs. resting state connectivity is that value-based decision-making is known to reliably induce variance in a striatal-limbic system [26], [45]. Across both delay and probability discounting trials, a main effect of group was observed, such that coupling between striatum and, among other regions, bilateral amygdala was significantly enhanced in gamblers compared to control subjects. This effect was similar for both left and right striatal seeds, and similar for delay and probability discounting conditions (i.e., no group x task interaction was observed).

Our results converge with previous findings of enhanced limbic connectivity in resting state analyses in other ICDs [27]–[29]. In healthy controls, striatal-amygdala functional connectivity is increased during highly rewarding situations, e.g. when listening to pleasurable music [46], but also more generally during the processing of salient stimuli [47]. Fronto-limbic connectivity was also increased in pathological computer game players in a cue reactivity study [48]. Interestingly, interactions between striatum and amygdala have been shown to underlie a range of reward-related behaviours in animal models. For example, input from the basolateral amygdala to the ventral striatum regulates reward-related striatal activity [32]. Coherence between striatum and amygdala is increased during the processing of reward-predictive cues [33] and inhibition of the amygdala-striatal projection reduces reward-seeking behaviour [34]. Taken together, these findings are compatible with the idea that impulsive behaviour (e.g. in ICDs) may be in part driven by increases functional coupling in networks that regulate reward-guided behaviour in animal models.

Self-control refers to the ability to overcome the urge to select tempting but ultimately inferior decision options, e.g. smaller-sooner rewards during delay discounting, or larger rewards that have a very low probability in probability discounting tasks. Pathological gamblers show impairments in tasks that require self-control [7]–[10], [12]. This was also observed in the present dataset [11] although the effect of increased risk-taking was only a statistical trend. A prominent neural model suggests that self-control may occur through top-down control of limbic regions via the (lateral) prefrontal cortex [49]–[51]. Increased coherence among these limbic regions may reduce the ability of the PFC to exert top-down modulatory control, and may thus constitute a potential bottom-up mechanism driving impulsive behaviour.

How may group differences in functional connectivity arise? One possibility is that they arise from group differences in anatomical connectivity. For example, in healthy subjects, the degree of amygdala-striatal anatomical connectivity, estimated using diffusion tensor imaging (DTI) based probabilistic tractography, predicts functional connectivity between these regions [52]. Greater anatomical connectivity between striatum and amygdala is also associated with increased novelty seeking [53], a typical personality correlate of pathological gambling [54]–[56]. Future studies may further explore links between these domains by acquiring both structural and functional connectivity data in patients suffering from ICDs.

Our analyses did not reveal correlations with behavioural markers or gambling severity, but gamblers tended to be overall more impulsive in both experimental conditions (i.e., the impact of delay was enhanced, whereas the impact of probability tended to be attenuated). How modulations in task-related (or parametric) neural responses relate to the presently observed modulations in functional connectivity in gamblers remains unclear. We previously observed both increases and decreases in striatal value coding in gamblers, depending on the task [11], whereas the present data show elevated striatal-amygdala coupling across both tasks. Striatal-amygdala coupling therefore does not simply enhance or attenuate striatal value coding, but how these effects relate to each other (if at all) remains to be clarified. One possibility is that parametric striatal value coding may not be directly related to impulsive behaviour, but may rather reflect the degree of gambling-relatedness of a task. In contrast, elevated striatal-amygdala coupling may impair the ability of the prefrontal cortex to exert top-down control (see above), which would be in line with the tendency of gamblers to be more impulsive in both the delay and probability discounting conditions. Still, the fact that we did not observe correlations between behavioural impulsivity and striatal-amygdala coupling argues against this.

It is possible that larger samples may be required to reveal an association between gambling severity and the present functional connectivity measures. On the other hand, previous studies from our lab have revealed reliable associations between gambling severity and neural effects [11], [15]. However, functional connectivity differences between controls and gamblers may be of a more categorical nature than these task-related responses. Connectivity differences might also reflect differences in task engagement between groups, although reaction times were not different between groups, which might argue against this. Future studies may benefit from the use autonomic measures to control for e.g. arousal. Nonetheless, it is possible that tasks such as these may generally be more rewarding for gamblers, an interesting possibility that would be in line with recent data showing a relative over-valuation of monetary vs. primary rewards in gamblers [19].

A shortcoming of our cross-sectional approach is that our data cannot reveal whether the observed increases in connectivity are the cause or the consequence of PG. However, on-going longitudinal studies such as IMAGEN [57] will hopefully shed light on whether increases in connectivity are a cause or a consequence of ICDs. Finally, unlike previous studies in other ICDs, we did not examine the resting state [27]–[29] but connectivity during a value-based choice task (although this is not strictly speaking a limitation). Future studies are therefore required to establish whether the present findings extend to the resting state.

Taken together, our data reveal a reliable enhancement of striatal-amygdala functional interactions in pathological gamblers during value-based decision-making. Interactions between these regions have been implicated in other ICDs, as well as in animal models of reward-guided behaviour. Our results in pathological gamblers therefore add to increasing evidence that elevated connectivity in limbic circuits may contribute to both substance-based [28], [29] and behavioural ICDs [27].
